# The use of interim data and Data Monitoring Committee recommendations in randomized controlled trial reports: frequency, implications and potential sources of bias

**DOI:** 10.1186/1471-2288-8-12

**Published:** 2008-03-20

**Authors:** Puvan Tharmanathan, Melanie Calvert, John Hampton, Nick Freemantle

**Affiliations:** 1 Department of Primary Care and General Practice, University of Birmingham, Birmingham, UK; 2 Division of Cardiovascular Medicine, Queen's Medical Centre, Nottingham, UK

## Abstract

**Background:**

Interim analysis of accumulating trial data is important to protect participant safety during randomized controlled trials (RCTs). Data Monitoring Committees (DMCs) often undertake such analyses, but their widening role may lead to extended use of interim analysis or recommendations that could potentially bias trial results.

**Methods:**

Systematic search of eight major publications: *Annals of Internal Medicine, BMJ, Circulation, CID, JAMA, JCO, Lancet *and *NEJM*, including all randomised controlled trials (RCTs) between June 2000 and May 2005 to identify RCTs that reported use of interim analysis, with or without DMC involvement. Recommendations made by the DMC or based on interim analysis were identified and potential sources of bias assessed. Independent double data extraction was performed on all included trials.

**Results:**

We identified 1772 RCTs, of which 470 (27%; 470/1772) reported the use of a DMC and a further 116 (7%; 116/1772) trials reported some form of interim analysis without explicit mention of a DMC. There were 28 trials (24 with a formal DMC), randomizing a total of 79396 participants, identified as recommending changes to the trial that may have lead to biased results. In most of these, some form of sample size re-estimation was recommended with four trials also reporting changes to trial endpoints. The review relied on information reported in the primary publications and methods papers relating to the trials, higher rates of use may have occurred but not been reported.

**Conclusion:**

The reported use of interim analysis and DMCs in clinical trials has been increasing in recent years. It is reassuring that in most cases recommendations were made in the interest of participant safety. However, in practice, recommendations that may lead to potentially biased trial results are being made.

## Background

Randomized controlled trials (RCTs) provide essential evidence on the efficacy and safety of medical interventions. Monitoring of accruing trial data is important to ensure participant safety [1,2]. The International Conference on Harmonisation (ICH) recommends that such analyses be conducted by individuals with no vested interest in the outcome of the trial where possible, particularly in large confirmatory Phase III trials [3].

When formalized, this role is commonly performed by a multi-disciplinary group of specialist, often referred to as a Data Monitoring Committee (DMC), which makes recommendations to those in charge of the overall management of the trial, often referred to as the Trial Steering Committee (TSC). Also, interim analyses should preferably be pre-specified in the trial protocol to minimize bias from unplanned analyses [1]. DMC recommendations typically include either: continuing the trial as planned; stopping early for hazard; stopping because efficacy is unequivocally established; or stopping because continuing the trial is futile [4].

Whilst it is widely accepted that the primary role of the DMC is to protect participant safety, several other roles of the DMC have been suggested, including recommending protocol amendments based on interim analysis [5]. Such amendments can lead to potentially biased results and damage the integrity of the trial as highlighted in recent FDA guidance:

*"Many kinds of trial modifications (e.g. changing endpoints, changing or adding to pre-specified analysis subgroups) could, if made with knowledge of trial results, have significant effects on type I error and interpretation of final results. If it is perceived that emerging results could have influenced these types of interim protocol changes, the credibility of the trial may be severely damaged. In general, to minimize the potential for bias, the trial leadership, which is insulated from knowledge of the interim data, rather than the DMC, should be responsible for proposing potential changes other than those driven by safety considerations." *[1]

It is unclear to what extent protocol modifications have been made in trials based on interim analyses, and more specifically as a result of recommendations by DMCs. Therefore, we systematically reviewed the reported use of interim analyses and DMCs to identify trials in which interim data have been used for reasons other than to make one of the recommendation described above, referring to these occurrences as "extended use of interim analysis" or "extended recommendations by DMCs" for the purposes of discussion here. Patterns of interim analysis use, roles assumed by the DMCs, and potential bias-related and ethical issues arising from such actions are discussed.

## Methods

### Data Sources and Searches

A search strategy that allowed for the identification of relevant case studies with extended recommendations based on interim analysis or by DMC was used. We conducted a systematic search of eight journals, *Annals of Internal Medicine, BMJ, Circulation, Clinical Infectious Diseases (CID), JAMA, the Journal of Clinical Oncology (JCO), Lancet *and *the New England Journal of Medicine (NEJM)*, to identify RCTs reporting the use of interim analysis with or without specification of a DMC, between June 2000 and June 2005. These journals were selected to include major generalist and specialist medical journals.

The title and abstract of every citation in the full-text journal content databases published within the time period searched was checked to identify whether it was indeed a main results paper of a RCT. Subsequently, the full-text of all RCT articles in the Portable Document Format (PDF) was scanned using a set of keywords. These keywords were found to be most likely to appear in relevant sections of the articles during a piloting exercise. This included *interim, monitor, early, stop, terminate, survival, death *and *mortality*.

### Study Selection and Data Extraction

Identified RCTs were classified according to therapeutic area and assessed (by P.T.) to determine: whether mortality was listed as an endpoint (primary, secondary or part of a composite endpoint), whether interim analysis was reported, and whether a DMC was reported. Various names referring to DMCs were considered.

All trials reporting interim analysis were categorized, based on trial progress or outcome and therefore classified as either 'Continued to or Extended beyond planned conclusion', 'Early stop for benefit', 'Early stop for harm', 'Stopped for futility' or 'Early stop for other reasons'. Next, using information in the results and where available design articles or trial protocols, trials were assessed to determine whether other actions of the DMC or release of interim data could have led to "extended recommendations" and whether these had the potential to introduce bias or complicate the interpretation of the results.

Independent double data extraction (M.C., P.T.) was performed on all trials considered to have "extended" use of interim data or DMC recommendations. A non-linear model accounting for journal was used to estimate whether the rate of DMC use increased by year.

## Results

### Reported use of Interim analysis and DMCs

In total, 1772 trials were identified, of which 470 (27%; 470/1772) reported the use of interim analysis and specified use of a DMC (this count includes trials that reported use of a DMC and interim monitoring plans, but did not report implementation of the analysis during the course of the trial). A further 116 trials reported some form of interim analysis without explicit mention of a DMC, giving a total of 586 (33%; 586/1772) trials that reported the use of interim analysis. There was a higher rate of reported use in trials that included mortality as an endpoint (54%; 410/764). The *Lancet *published most trials (352/1682; 21%), with *JCO *reporting the most mortality trials (233/285; 82%).

The proportion of trial reports describing the use of interim analysis, with or without specification of a DMC, ranged from 10% (21/209) in the *BMJ *to 58% (160/274) in the *NEJM*. The reported use in mortality trials also varied with the *JCO *reporting interim analysis in 35% (81/233) of such trials compared to over 70% in *JAMA *(44/69),*NEJM *(112/149) and the *Lancet *(94/130).

The reported use within specific therapeutic areas ranged from 30% (22/71) in Pediatrics to 47% (20/43) in Obstetrics and Gynecology (Table 1).

**Table 1:** Breakdown of retrieved trials by Therapeutic Area

**Therapeutic Area**	**Total number of trials retrieved in search**	**Trials reporting interim analysis (with or without specification of a DMC)**	**Trials identified as having extended use of interim data or DMC recommendations with the potential to introduce bias**
**Total (N)**	**1772**	**586**	**28**
Cardiovascular Disease (CVD)	494	209	11
Oncology (ONC)	387	136	11
Infection (INF)	271	93	2
Pediatrics (PED)	71	22	0
Obstetrics & Gynecology (OBGYN)	43	20	2
Neurology (NEURO)	50	17	1
Other	456	89	1

There was a significant (P < .001) rise in the number of trials reporting use of interim analysis over the period reviewed, with 86 trials reporting use between June 2000 to May 2001, and 151 trials for June 2004 to May 2005.

### Recommendations based on interim analysis or made by DMCs

Of the 586 trials that reported use of interim analysis, with or without specification of a DMC, 444 trials (76%; 444/586) continued as planned, 75 trials (13%; 75/586) stopped early because of benefit or harm to study participants, 26 (4%; 26/586) stopped early for other reasons and 28 (5%; 28/586) were stopped for futility (Table 2).

**Table 2:** Overall classification of RCTs reporting interim analysis or DMC use based on trial progress

**Trial Progress**	**Trials reporting interim analysis (with or without specification of a DMC)**	**Trials identified as having extended use of interim data or DMC recommendations**	**Trials identified as having extended use of interim data or DMC recommendations with the potential to introduce bias**
**Total (N)**	**586**	**74**	**28**
Continued to or Extended beyond planned conclusion	457	55	26
Early stop for benefit	53	7	1
Early stop for harm	22	3	0
Stopped for futility	28	7	0
Early stop for other reasons	26	2	1

**Figure 1:**
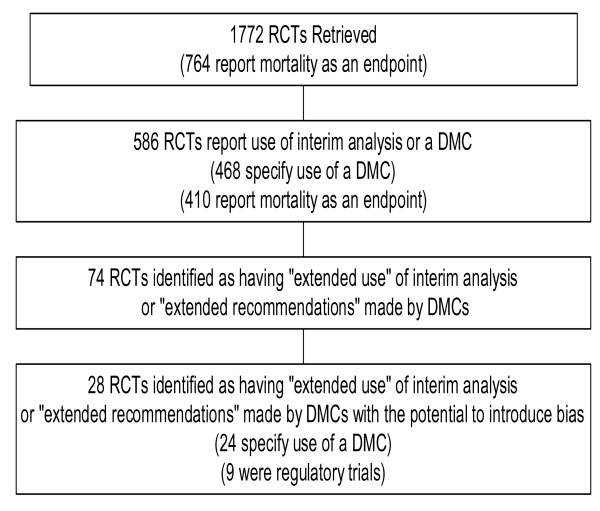
Identification of Randomized Controlled Trials retrieved during Systematic Search.

A total of 74 trial reports (13%; 74/586) had "extended" use or recommendations and were further examined (Figure 1, Table 2). Of these, 46 (8%; 46/586) trials included recommendations or amendments aiming to ensure participant safety, which is unlikely to have introduced bias, and therefore did not complicate the interpretation of the results. For example, the expansion of exclusion criteria to ensure that children with unrecognized HIV infections were not included was recommended by the DMC subsequent to interim analysis in a trial comparing the use of oral amoxicillin with injectable penicillin for severe pneumonia [6].

There were 28 trials (5%; 28/586), randomizing 79396 participants, identified as having extended recommendations based on interim analysis with the potential to introduce bias to trial results, or to lead to problems with interpretation. Most were either cardiovascular or oncology trials and the extended use or recommendations noted in these trials are detailed in Additional file 1.

Among these, 24 trials (randomizing a total of 75838 participants) reported use of DMCs while there was reported use of interim analysis without specific mention of DMCs in the remaining four studies. Nine of these 28 trials were regulatory trials (Figure 1).

### Trials identified as having extended use of interim data or DMC recommendations with the potential to introduce bias

#### Sample size re-estimation (SSR)

In 27 of the 28 trials that were judged to be open to bias, some form of SSR was recommended subsequent to the review of interim data. The recommendation for SSR was implemented as a protocol amendment in 26 of these (see Additional file 1).

Several publications reported that interim trends observed in individual treatment arms were used as the basis for a SSR recommendation [7-12]. For instance, the Public-Access Defibrillation and Survival after Out-Of-Hospital Cardiac Arrest (PAD) trial [12], assessed whether training laypersons to use automated external defibrillators in addition to cardiopulmonary resuscitation (CPR) increased survival from out of hospital cardiac arrest. The report states that '(T)he observed numbers of arrests were substantially lower than anticipated from the pre-randomization unit-enrolment data; however, the survival rate in the CPR-only units was higher than anticipated. After the interim analysis, information regarding the frequency of cardiac arrests and the survival rate in the CPR-only units was used to extend the data-collection period by six months to maintain the specified power level'.

There also were examples where the authors were specific that the SSR recommendation was made subsequent to interim analysis or by the DMC based on overall rates [13-18]. In six trials where SSR was recommended, the authors make reference to the use of masked interim data [13,15,16,19-21]. In these cases, data reviewed by the DMC did not identify which treatment was associated with each group.

#### Trial endpoint amendments

Two trials reported changes to the primary endpoint based on interim analysis [9,15], one introduced an additional (secondary) endpoint [22] and one dropped an endpoint [23] subsequent to interim analyses.

CAPRICORN (CArvedilol PostinfaRct survIval COntRol in LV dysfunctioN) investigated the effects of carvedilol, a beta-blocker, in patients with left ventricular dysfunction following myocardial infarction [15]. The original primary endpoint in the protocol was all-cause mortality. However, while the study was ongoing, the primary endpoint was amended by the TSC by dividing the available statistical power (P = 0.05) between a new composite endpoint (all-cause mortality or cardiovascular hospital admissions, P = 0.045) and the original primary endpoint (all-cause mortality, P = 0.005). The results publication for this trial states that the TSC took the decision because the DMC 'noted that overall mortality was lower than had been predicted and that the study could not be completed with the sample size and power originally planned.'

In a trial in chronic lymphatic leukemia that compared fludarabine with chlorambucil, the initial sample size was based on a comparison of the rates of complete remission [9]. However, there was a post-hoc change of the primary endpoint to progression-free survival, a time-to-event measure. The reason given for this change was 'that the response rate in the chlorambucil group was significantly lower than the rates in the other two groups,' as observed during an interim analysis.

## Discussion

The use of interim analysis and DMCs in clinical trials reported in major journals has increased in recent years. A possible explanation for the variation in the rate of reported use of interim analysis or DMCs among the journals searched (Table 1) is the nature of trials published and the methods of reporting in the publication. For instance, the high rate among trials published in the NEJM is probably because it often attracts landmark clinical trials that may have a higher likelihood of having used interim analysis and it was easier to spot relevant information due to the more structured format used by the journal.

Also of note is the large proportion of trials that included mortality as an endpoint in the JCO but comparatively low rate of interim analysis and DMC use among the trials published in that journal, possibly indicating a need for increased uptake or improved reporting in the oncology sector.

The majority of recommendations based on interim analysis or by DMCs were either to continue the trial as planned or to terminate early. This is reassuring as DMCs can be seen to be fulfilling their primary role of protecting participant safety. Our review also shows that, in practice, extended use of interim analysis or extended DMC recommendations are being made, some of which could potentially introduce bias and produce disputable results.

## Limitations

A limitation of our review is that we relied on information reported in the primary publications and methods papers relating to the trials, therefore trial publications that did not acknowledge the use of interim analysis or DMC and related protocol amendments might not have been identified. Furthermore, many trials that are terminated early or amended during the course of the trial may never be published.

### Sample size re-estimation (SSR) based on interim analysis or made by DMCs as a source of bias

The amendments identified were mostly sample size re-estimations due to insufficient accrual of participants or events. Power calculations are made based on assumptions that may not be reflected in the actual experience of a trial [24]. SSR is typically an attempt to ensure that a study's objective is accomplished with adequate power. SSR may also be necessary due to safety considerations, for example when a comparison in the trial is dropped for safety reasons or when new data from other sources becomes available during the progress of a trial.

A SSR made by the TSC based on overall event rates, without knowledge of the rates of events in specific groups, is not contentious and may be considered good practice. However, a recommendation for SSR made with knowledge of interim data introduces bias to the trial. For example, if SSR is undertaken based on a comparison of event rates observed during an interim analysis or if an SSR is conducted because of a lower than expected event rate in the control group. The latter introduces bias because rates in one group are integral to the result of the difference between the groups; this is a special case of regression to the mean [25].

In PAD [12], the CPR-only units formed the control group, and making changes to the protocol based on endpoint data from this group clearly has the potential to introduce bias.

In the trials where SSR was performed subsequent to interim analysis based on overall rates or the review of masked data, it would seem that the investigators were on the whole attempting to be explicit that the SSR recommendation was made without introducing bias. However, whether in all these cases the DMC were truly blind to treatment allocation is debatable since in the former, DMCs often also have access to treatment arm-specific rates and in the latter, differences in adverse events or trends in data may have inadvertently led to unmasking.

A trial examining whether low-dose dopamine attenuates renal injury as compared to placebo, where masking was implemented during the interim analysis [20], provides an example of the difficulties with reviewing masked data, since as the trialists described: 'there is concern about the potential adverse effects of low-dose dopamine on pituitary function, T-cell responsiveness, and gastrointestinal oxygenation'. These adverse events would not occur with any discernable frequency in the placebo arm, and would unmask a DMC with knowledge of them.

### Trial endpoint amendments based on interim analysis or made by DMCs as a source of bias

The primary endpoint *should be the variable capable of providing the most clinically relevant and convincing evidence directly related to the primary objective of the trial *[26]. Changing the primary endpoint of a study in the knowledge of the interim results is analogous to changing a bet to the winning horse part way through a race, introduces bias and leads to complications with interpretation of the final results.

In the cases where changes were made to the primary endpoint [9,15], it appears that these may have been attempts to achieve a positive result. In CAPRICORN, the introduction of a composite co-primary outcome subsequent to review of interim data can be seen as an attempt to achieve a positive trial by using a composite endpoint which would have a higher likelihood of accruing events. The original primary endpoint (all-cause mortality) achieved a P-value of 0.03 (i.e. substantially larger than the 0.005 subsequently allocated to it) whilst the composite endpoint of all-cause mortality and cardiovascular hospital admissions (new co-primary outcome) had a P-value of 0.30. Therefore, CAPRICORN should be considered a neutral trial as neither of the co-primary endpoints achieved statistical significance. Had the original primary endpoint of all-cause mortality been maintained, it would have resulted in a modestly statistically significant result and consequently CAPRICORN would have been considered a positive trial.

In the leukemia trial [9], multiple amendments, most notably amending the primary endpoint, were made subsequent to interim analysis with knowledge of treatment-arm specific rates in an apparent attempt to increase the number of events accrued and achieve a successful trial, but a substantial bias was introduced. The reporting in this trial also highlights the need for clarity when communicating final results subsequent to protocol amendments. The result of the original primary endpoint was found to be statistically significant and the manner in which the results of the trial were then reported in the publication obfuscates the endpoint amendments. Indeed, in the results section, the original primary endpoint was referred to first in the text.

## Conclusion

Our review of published trials indicates that the reporting of DMCs and interim analyses use in major journals has increased in recent years. Many trials, including those with mortality as an endpoint, did not appear to have any formal interim analysis, which is inadequate if we are to protect participant safety. In the majority of cases, recommendations were made based on interim analysis in the interest of participant safety and it is reassuring that there was only a small proportion of trials where extended use of interim analysis or recommendations made by the DMC had the potential to introduce bias.

However, these trials involved nearly eighty thousand participants and most had formal DMCs. Many trials that stop early may never be published or are published in relatively obscure journals [27]. Thus, the trials identified here are probably a subset of a larger group of trials that have even less adequately reported changes in design.

Empirical evidence has shown that trialists can attempt to achieve a positive trial using multiple strategies and this is a very real concern [28]. This review adds to these concerns by highlighting how interim data can be used to influence the results of a trial. There is clearly a need to ensure that interim data are not misused to this end. The role of DMCs, with their privileged access to interim data, must be carefully considered if bias is to be avoided.

## Competing interests

PT and MC have received research funding and consultancy fees from the pharmaceutical and medical device industry. JH and NF have received funding from a range of sources including the pharmaceutical industry, research charities and governmental bodies to undertake a number of roles in the design, conduct and interpretation of randomized trials.

## Authors' contributions

PT conducted the systematic literature review, selection and categorization of cases, data extraction, data analysis, writing of the manuscript, and is guarantor. MC conducted data extraction, data analysis, and writing of the manuscript. NF contributed to the data extraction, data analysis and writing of the manuscript. JH contributed to the development of the manuscript.

## Pre-publication history

The pre-publication history for this paper can be accessed here:



## Supplementary Material

Additional file 1 RCTs identified as having extended use of interim analysis or extended recommendations made by DMCs, with potential to introduce bias. A list of trials identified as having reported extended use of interim analysis or recommendations by DMCs with the potential to introduce bias. Trials are sorted based on the interim trends observed or reason for interim analysis related recommendation. ^ CVD-Cardiovascular Disease, INF-Infection, OBGYN-Obstetrics and Gynecology, ONC-Oncology, **NEURO**-Neurology. Information in brackets:. * R denotes a regulatory trial or that the intervention was investigational when the trial was completed and **DMC **denotes specification of a DMC in the trial publication. DMC specified in 24/28 cases, R trials: 9/28 cases. ** **TTE **denotes time to event. *** Information in brackets: **TA **denotes that treatment arm specific rate were analyzed during interim analysis, **OA **denotes that overall rates were analyzed during interim analysis, **Masked **denotes that masking of data was maintained during interim analysis (where specified/reported in the trial publication). Click here for file
