# Supplementary material for: The use of interim data and Data Monitoring Committee recommendations in randomized controlled trial reports: frequency, implications and potential sources of bias
Source: BMC Med Res Methodol. 2008 Mar 20;8:12. doi: 10.1186/1471-2288-8-12 (PMC2279143; doi:10.1186/1471-2288-8-12)
Supplement: Additional file 1 — RCTs identified as having extended use of interim analysis or extended recommendations made by DMCs, with potential to introduce bias. A list of trials identified as having reported extended use of interim analysis or recommendations by DMCs with the potential to introduce bias. Trials are sorted based on the interim trends observed or reason for interim analysis related recommendation. ^ CVD-Cardiovascular Disease, INF-Infection, OBGYN-Obstetrics and Gynecology, ONC-Oncology, NEURO-Neurology. Information in brackets:. * R denotes a regulatory trial or that the intervention was investigational when the trial was completed and DMC denotes specification of a DMC in the trial publication. DMC specified in 24/28 cases, R trials: 9/28 cases. ** TTE denotes time to event. *** Information in brackets: TA denotes that treatment arm specific rate were analyzed during interim analysis, OA denotes that overall rates were analyzed during interim analysis, Masked denotes that masking of data was maintained during interim analysis (where specified/reported in the trial publication). [file 1471-2288-8-12-S1.doc]

# **Additional file 1: RCTs identified as having extended use of interim analysis or extended recommendations made by DMCs with potential to introduce bias**

| **Source** | **Population size (N)** | **Therapeutic Area ^*** | **Extended use of interim analysis or extended recommendations made by DMC** | | | **Interim trend observed and/or Reason for interim analysis related recommendation**  ******* | **Primary Endpoint Type **** | **Was the trial reported as a positive trial (on the primary endpoint)?** |
| --- | --- | --- | --- | --- | --- | --- | --- | --- |
|  |  |  | **Sample Size Re-estimation** | | **Trial endpoint/s (primary/other) amended** |  |  |  |
|  |  |  | **Sample size amended** | **Extension of follow-up** |  |  |  |  |
| Rai et al. 2000 [9] | 509 | ONC (R) | Y (increase) | N | Y (primary) | Interim Trends (TA) | Counts amended to TTE | Y (on new)  Y (on original) |
| To et al. 2004 [10] | 253 | OBGYN (DMC) | Y (increase) | N | N | Interim Trends (TA) | Dichotomous | N |
| Hersey et al. 2002 [11] | 700 | ONC (R) | Y (increase) | N | N | Interim Trends (TA) | TTE | N |
| CARE-HF [7] | 813 | CVD (R/DMC) | N | Y (not as suggested by DMC) | N | Interim trends (TA) | TTE | Y* |
| PAD [12] | 19000 | CVD (DMC) | N | Y | N | Interim Trends (TA) | Counts | Y |
| ICON4/AGO-OVAR-2.2 [8] | 802 | ONC (DMC) | N | N | N | Interim Trends (TA) | TTE | Y |
| Kaul et al. 2004 [14] | 466 | INF (DMC) | Y (increase) | Y | N | Low event rate (OA) | Counts | N |
| Schmid et al. 2003 [17] | 2021 | ONC | Y (increase) | N | N | Interim trends (OA) | TTE | N |
| Kastrati et al. 2004 [18] | 2159 | CVD (DMC) | Y (increase) | N | N | Low event rate (OA) | Dichotomous | N |
| CAMELOT [29] | 1991 | CVD (DMC) | Y (reduction) | N | N | High event rate (OA) | TTE | Y |
| Chintu et al. 2004 [30] | 541 | INF (DMC) | Y (reduction) | N | N | High event rate and lower losses to follow-up (OA) | TTE | Y |
| SPORTIF [31] | 3922 | CVD (R/DMC) | N | Y | N | Low event rate (OA) | TTE | Y |
| CAPRICORN [15] | 1959 | CVD (R/DMC) | Y (increase) | N | Y (primary) | Interim trends (OA, Masked) | TTE | N (on new)  Y (on original) |
| MAGIC [19] | 6213 | CVD (DMC) | Y (reduction) | N | N | Interim trend (OA, Masked) | TTE | N |
| Holmberg et al. 2002 [13] | 695 | ONC (DMC) | Y (increase) | N | N | Low event rate (OA, Masked) | TTE | Y |
| (PARAGON)-B [16] | 5225 | CVD (R/DMC) | Y (increase) | N | N | Low event rate (OA, Masked) | Dichotomous | N |
| Mohr et al. 2001 [21] | 2206 | CVD (DMC) | Y (increase) | N | N | To adjust for effects of interruption of therapy  (OA, Masked) | TTE | N |
| ANZICS [20] | 328 | RENAL | Y (increase) | N | N | To increase statistical power (Masked) | Continuous | N |
| Movsas et al. 2005 [23] | 243 | ONC (DMC) | Y (reduction) | N | Y (other) | New external information | Dichotomous | N |
| INVEST [22] | 22576 | CVD (DMC) | Y (reduction) | N | Y (other) | High event rate & external information | TTE | N |
| Kaiser et al. 2002 [32] | 312 | ONC (DMC) | Y (increase) | N | N | Interim Trends and conditional power | TTE | N |
| Kupperman et al. 2004 [33] | 63 | OBGYN (DMC) | Y (reduction) | N | N | Poor recruitment | Continuous | Y |
| Hanks et al 2003 [34] | 1554 | ONC (R/DMC) | Y (increase) | N | N | Additional sub-group analysis | TTE | Y |
| Lees et al 2000 [35] | 1804 | NEURO (R/DMC) | Y (increase) | N | N | An unexpectedly high primary haemorrhage rate | Dichotomous | N |
| Rudd et al. 2005 [36] | 422 | ONC (R /DMC) | Y (increase) | N | N | Not given | TTE | Y |
| Andrews et al. 2004 [37] | 333 | ONC (DMC) | Y (increase) | N | N | Fewer patients than planned received treatment | TTE | N |
| Reyes et al. 2005 [38] | 647 | ONC (DMC) | Y (increase) | N | N | Effect size amended. | TTE | Y |
| (ASSENT)-3 PLUS [39] | 1639 | CVD (DMC) | Y (increase) | N | N | Interim trends used to recommend extension of a feasibility study | Dichotomous | N |
